# Examining Neural Plasticity for Slip-Perturbation Training: An fMRI Study

**DOI:** 10.3389/fneur.2018.01181

**Published:** 2019-01-23

**Authors:** Prakruti J. Patel, Tanvi Bhatt, Sophie R. DelDonno, Scott A. Langenecker, Shamali Dusane

**Affiliations:** ^1^Cognitive-Motor and Balance Rehabilitation Laboratory, Department of Physical Therapy, College of Applied Health Sciences, University of Illinois at Chicago, Chicago, IL, United States; ^2^Department of Psychiatry, College of Medicine, University of Illinois at Chicago, Chicago, IL, United States

**Keywords:** fMRI, pertubation, balance, stability, cortex

## Abstract

Perturbation-based balance training has shown to induce adaptation of reactive balance responses that can significantly reduce longer-term fall risk in older adults. While specific cortical and subcortical areas in control of posture and locomotion have been identified, little is known about the training-induced plasticity occurring in neural substrates for challenging tasks involving reactive balance control. The purpose of this study was to use functional neuroimaging to examine and determine the neural substrates, if any, involved in inducing adaptation to slip-like perturbations experienced during walking over 3 consecutive training days. We used a mental imagery task to examine the neural changes accompanied by treadmill-slip perturbation training. Ten healthy young adults were exposed to increasing magnitude of displacements during slip-like perturbations while walking, with an acceleration of 6 m/s^2^ on a motorized treadmill for 3 consecutive days. Brain activity was recorded through MRI while performing imagined slipping and imagined walking tasks before and after the perturbation training. The number of compensatory steps and center of mass state stability at compensatory step touchdown were recorded. As compared with day 1 (first trial), on day 3 (last trial) there was a significant reduction in number of compensatory steps and increase in stability at compensatory step touchdown on the mid and highest perturbation intensities. Before perturbation training, imagined slipping showed increased activity in the SMA, parietal regions, parahippocampal gyrus, and cingulate gyrus compared with rest. After perturbation training, imagined slipping showed increased activation in DLPFC, superior parietal lobule, inferior occipital gyrus, and lingual gyrus. Perturbation training was not associated with decline in activity in any of the brain regions. This study provides evidence for learning-related changes in cortical structures while adapting to slip-like perturbations while walking. The findings reflect that higher-level processing is required for timing and sequencing of movements to execute an effective balance response to perturbations. Specifically, the CNS relies on DLPFC along with motor, parietal, and occipital cortices for adapting to postural tasks posing a significant threat to balance.

## Introduction

The central nervous system (CNS) possesses the ability to adapt to novel sensorimotor stimuli. With regards to controlling stability during dynamic balance tasks, the adaptive capacity of CNS has been studied by exposing healthy younger and older adults to repeated slip-like perturbations (1, 2). This is also known as perturbation training, a novel paradigm for preventing falls while walking. During subject-controlled perturbations, i.e., overground slip-like perturbations, the CNS utilizes error information from initial perturbations to shift from a feedback or reactive control to a feedforward or proactive control to produce protective responses (1, 3). Feedforward adaptations are observed in the form of reduced ankle dorsiflexion, increased knee flexion, and reduced heel contact velocity of the slipping leg which influence the ground reaction forces resulting in a reduced slip-perturbation intensity (4–7). On the other hand, during experimenter controlled perturbations, CNS relies predominantly on feedback system, for example, increasing compensatory step length to maintain a more forward center of mass state (position and velocity) at step completion, thus achieving a more stable position (8, 9). These studies provide a substantial understanding about the behavioral mechanisms involved in adaptation to perturbations however, the neural mechanisms underlying such adaptions remain unclear and largely speculative.

Extensive research has identified the widespread neural networks engaged in regular locomotion by examining both mental imagery of walking via functional magnetic resonance imagining (fMRI), and real walking through single-photon-emission-computed-tomography (SPECT) and positron-emission tomography (10, 11). While there is strong evidence for cortical and subcortical control of steady state walking (10–13), other studies show that a specific neural activation pattern is involved in challenging walking tasks. A recent study using high-density EEG identified cortical activity related to loss of balance while walking on a balance beam mounted on a treadmill. Interestingly, they found an increase in theta band spectral power in anterior cingulate, posterior cingulate, anterior parietal, sensorimotor and dorsolateral-prefrontal cortices exclusively during loss of balance from the balance beam (14). Another study using functional MRI reported modulation of brain activity, specifically in bilateral superior parietal lobule and middle occipital gyrus with mental imagery of walking on a narrow path compared with a wider path (15). The above studies suggest that maintaining balance in presence of task constraints is associated with different neural activation pattern than regular walking.

Neural adaptations to improvement in posture control has been examined predominantly during standing balance training through modulation of H-reflex responses (16). Many studies report down regulation of the spinal, H-reflex following single and multiple sessions of balance training (17, 18). For example, Trimble and Koceja reported a 26.2% reduction in the H-reflex amplitude post-training compared with pre-training (18). Similarly, Mynark and Koceja reported a reduction in H-reflex relative to background muscle activity after 2 days of balance training which related with reduced body sway while standing (19). It is suggested that adaptation to balance tasks through reduced modulation of H-reflex is accompanied by greater influence of the supraspinal mechanisms for balance control (20). While these studies provide evidence for a possible supraspinal modulation with improved balance control, the specific structures involved in adaptation to balance tasks are not known.

Balance recovery from external perturbations require rapid processing of sensorimotor information and execution of an accurate reactive or compensatory response (21). Walking involves constantly shifting balance between double and single-limb support phases while maintaining forward progression. Furthermore, external disturbance in balance can occur at any point in the gait cycle which increases the challenge for maintaining balance in case of an external disturbance. However, the neural changes involved in adaptation to external perturbations during walking are poorly understood. A few studies have examined corticomotor excitability before and after locomotor training using transcranial magnetic stimulation. Fisher et al. demonstrated an improvement in stride length and increased cortical silent period after high intensity locomotor training in people with Parkinson's disease, suggesting locomotor training alters the corticomotor activity (22). Furthermore, in stroke survivors, improvement in motor threshold (measured with TMS) following locomotor-balance training correlated with increased step length while walking (23). These studies support the view that locomotor training is related to modulation of cortical activity. It is therefore likely that perturbation based locomotor training could involve changes in neural activity.

Reactive balance responses are likely to engage brain areas related to evaluation of sensory information, development of a new motor plan or recalibration of an existing motor plan to carry out an appropriate action. Perturbation training studies (2, 24), neurophysiological studies (25, 26), and neuroimaging studies (27) support that reactive responses to large perturbations may be modulated through cortical regions particularly, motor cortex. For instance, Adkin et al. demonstrated a negative potential in the fronto-central cortical area occurring 100 ms after perturbation onset while standing (26). Such a response is linked with error detection (28), suggesting a role of cortical areas in modulating reactive balance responses. Although reactive responses involve long and short loop reflexes at spinal cord and brainstem levels (29–32), it is likely that initial adaptation to novel unexpected perturbations may involve cortical regions for error detection and feedback. Therefore, the purpose of this study was to identify the neural regions involved in adaptation to slip-like perturbations during walking. In particular, we were interested in identifying areas showing changes (increase or decrease) in activation during mental imagery of walking slip-perturbations after undergoing 3 consecutive days of treadmill-slip perturbation training among healthy young adults. Considering that slip perturbation was a novel stimulus during walking, we hypothesized that after training there would be increased activation in regions related to sensorimotor processing, balance control, sequence control, and memory.

## Methods

### Participants

**Figure 1:**
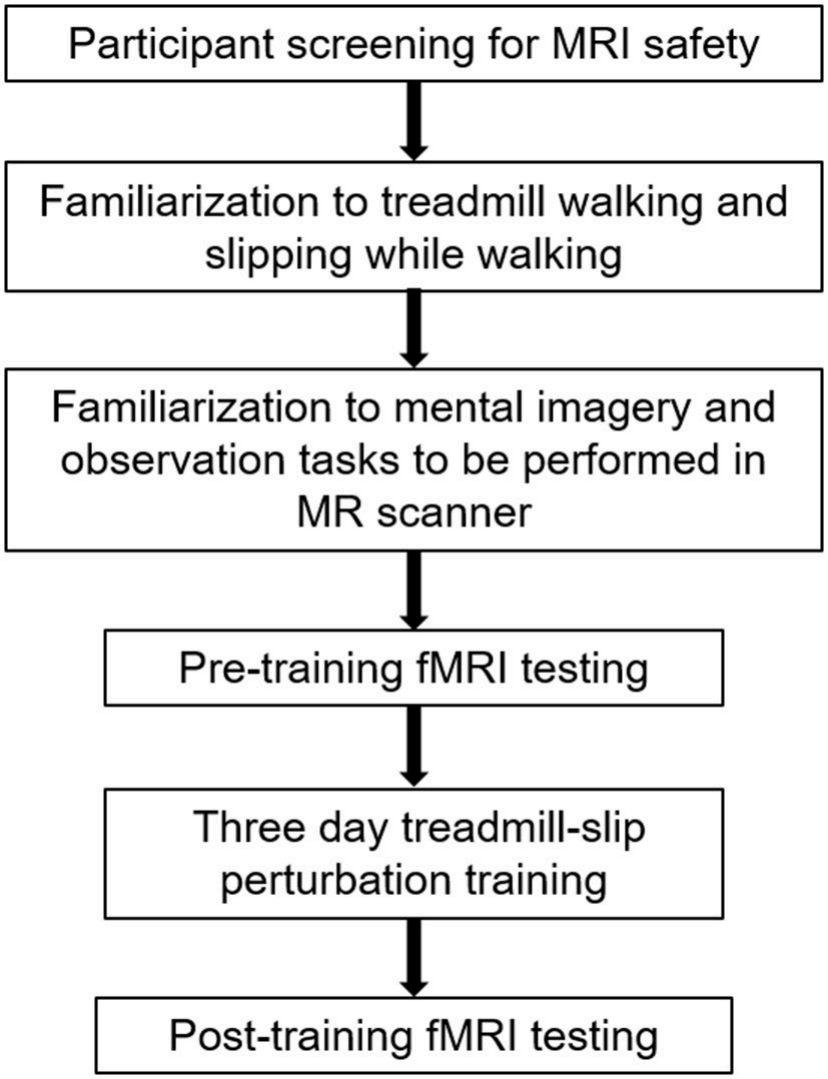
A schematic representation of the experimental protocol. The initial session in the experimental protocol comprised participant screening for fMRI (functional magnetic resonance imaging) safety, familiarization to treadmill, and fMRI tasks, and the first, pre-training fMRI scan. The familiarization and pre-training scan were performed on the same day. This was followed by treadmill slip-perturbation training for 3 consecutive days. The next day after perturbation training, we performed the post-training fMRI scan.

Ten young adults (27 ± 4 years; range 20–34 years) were enrolled into the study after obtaining a written informed consent approved by the Institutional Review Board. The participants were screened for magnetic resonance imaging (MRI) safety and any neurological, musculoskeletal or cardiovascular disorders. All participants underwent 3 days of treadmill-slip perturbation training while walking. They also performed mental imagery tasks in the MR scanner before and after perturbation training. Figure 1 shows a schematic presentation of the study protocol.

### Regular and Perturbed Walking Tasks

The regular and perturbed walking trials were performed before the first fMRI session (i.e., pre-training fMRI recording). In this session, all the participants were familiarized to regular walking on the treadmill and slipping while walking on the treadmill to facilitate mental imagery of these tasks in the MR scanner. A single regular walking and a single perturbed walking trial was performed. Participants stood at the center of the ActiveStep treadmill (Simbex, NH) with their feet shoulder width apart. All participants donned a safety harness which prevented their knees from touching the treadmill belt in case they experienced a fall. While standing on the treadmill, participants faced a window covered with blinds to prevent any distraction in treadmill tasks. At the start of every treadmill trial, participants were reminded to look straight at the window in front of them. The treadmill walking tasks comprised of—(1) regular walking and (2) experiencing a single unexpected slip i.e., backward loss of balance while walking. During regular walking, participants were instructed to walk naturally and the treadmill speed was adjusted to their preferred walking speed (0.9–1.2 m/s). Participants walked at their preferred walking speed for 1 minute. Following this, participants were instructed that they may experience a sudden slip-like perturbation at an unpredictable instant on the following walking trial. Participants were asked to recover their balance to the best of their ability. Participants were exposed to a single slip-like perturbation with an acceleration of 12.00 m/s^2^ (0.25 m displacement for 0.25 s). This perturbation intensity was chosen to provide a real-life like slipping and falling experience and to facilitate the mental imagery of slipping while walking in the MR scanner. During the walking tasks, participants were instructed to concentrate on the experience of walking on the treadmill with regards to movement of their body parts, differences in movement of the body segments and sensory stimuli experienced on the feet. The experimenter standing nearby provided verbal cues to assist the participants in noticing the body movements and sensations. The verbal cues included “focus on position of the different body segments” and “focus on the sensations on the feet.” The same researcher provided these verbal cues to each participant. This procedure was performed only before the pre-training fMRI session and not before the post-training fMRI session.

### Treadmill-Slip Perturbation Training

**Figure 2:**
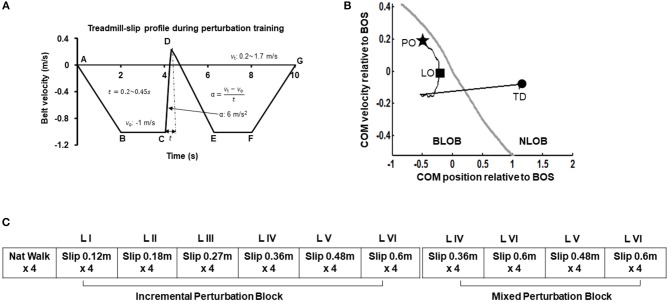
**(A)** Treadmill-slip perturbation training profile of level III perturbation intensity with an acceleration of 6 m/s^2^ at constant belt velocity of 1 m/s, and perturbation displacement and duration of 0.27 m and 0.3 s, respectively. Part A signifies the time instant at which the treadmill belt starts moving backwards and participant initiates walking. The treadmill belt backward velocity ramps up to −1 m/s (A to B), followed by a phase of constant velocity equal to the normal gait speed of healthy young adults (~1 m/s) (B to C). Part C signifies the time instant at which treadmill belt movement reverses in the forward direction to initiate a slip-like perturbation. The treadmill belt suddenly accelerates at a rate of 6 m/s^2^ (C to D), inducing a slip-like perturbation resulting in a backward loss of balance during walking. The treadmill belt then continues to move backward and accelerates (D to E) to reach the constant velocity allowing regular walking (E to F) followed by a gradual stop (F to G). *v*_o_, initial belt velocity in m/s; *v*_t_, terminal belt velocity in m/s; *t*, perturbation duration in seconds; α, belt acceleration in m/s^2^. The six different perturbation intensities were generated by modulating time (*t*) taken to achieve the final velocity (*v*_t_) at the constant acceleration of 6 m/s^2^. **(B)** Treadmill-slip perturbation training protocol for 3 consecutive days demonstrating an initial incremental (increasing intensity) perturbation training block followed by a mixed perturbation block comprising of different perturbation intensities. *Nat walk*, Natural walking without experiencing any perturbations; *LI to LVI*: The difference levels of perturbation training intensities. **(C)** A typical trajectory of center of mass state (COM position and velocity) stability relative to the most posterior margin of base of support (BOS) (black line) from perturbation onset (PO, star) until the completion of the recovery response i.e., compensatory step touchdown (TD, circle). The gray like represents the theoretical threshold for backward loss of balance (BLOB) (37). After treadmill-slip onset, the COM state stability transitions to a more posterior (negative) state at compensatory step liftoff (LO) causing BLOB and then shifts to a more anterior (positive) state as the BOS is re-established with a compensatory step leading to no loss of balance (NLOB).

Participants were informed that they may or may not experience sudden slip-like perturbations while walking on a treadmill without any prior indication. First all participants walked naturally on the treadmill. The treadmill speed was adjusted to match their self-selected walking speed (0.9–1.2 m/s). After four natural walking trials, on the fifth walking trial, participants were exposed to a slip-like perturbation on the treadmill at an unexpected time. Participants were asked to perform a natural response to recover balance to the best of their ability and continue walking on the treadmill. The very first treadmill-slip perturbation was delivered at the lowest intensity (level I, distance 0.12 m for 0.2 s and acceleration 6 m/s^2^). Further, participants were subjected to three more perturbation trials at the same intensity. If the participant successfully recovered from the treadmill-slips on three out of four perturbation trials (i.e., no falls) at a particular perturbation intensity, they were exposed to four additional treadmill-slip trials on the next higher perturbation intensity (level II, distance 0.18 m for 0.25 s and acceleration 6 m/s^2^) wherein they were again exposed to four consecutive treadmill-slip perturbations. Following consecutive recoveries, participants were subjected to higher intensities of treadmill-slips up to the highest perturbation intensity (level VI) (Figure 2A). This comprised of the incremental treadmill-slip perturbation training block. After the incremental treadmill-slip perturbation training, participants were exposed to mixed treadmill-slip perturbation training block at levels IV–VI (Figure 2B). If the participant had a fall on any of the treadmill-slip trials in a given block, the perturbation intensity was not progressed to the higher level and the training continued at the same perturbation intensity followed by a mixed block of perturbations at the level where the participant fell and its consecutive next lower intensity. The same procedure was followed for 3 consecutive days of treadmill-slip perturbation training.

In each training session, all walking trials (perturbation and regular) lasted for 10 s. At the beginning of training session, participants were informed that they may or may not receive a perturbation on the following walking trials. In the perturbation trials, the perturbation onset was set to occur after initial 3–7 steps to reduce predictability of the perturbation. While the participants were aware that they would be exposed to several perturbed walking trials, they were unaware that they would be exposed to different perturbation intensities. The training perturbation intensity (acceleration and displacement) was selected such that it would sufficiently challenge participant's reactive balance as well as provide opportunity to improve reactive balance through training. Further, the training perturbation intensity was also chosen based on our previous study demonstrating that treadmill-based slip perturbation training at 5–6 m/s^2^ with displacements 0.1 to 0.9 m improves reactive balance on novel overground slips (33).

### fMRI Tasks

The fMRI sessions were conducted 3–4 days before the first treadmill-slip training session and immediately after the third training session on the same day. fMRI is a non-invasive functional neuroimaging technique wherein activation of brain areas is tracked by detecting the change in blood flow using the blood oxygen level dependent (BOLD) contrast. It is a widely used technique to map the brain regions linked with functions such as movement, speech, and cognition. Prior to each MR session, participants were trained on the mental imagery of the experimental tasks, instructing them to rely upon their experience of walking and slipping on the treadmill. Their ability to form a mental imagery was assessed with Vividness of Visual Imagery Questionnaire. During the observation tasks, participants were instructed to observe a video of another person walking, or slipping while walking on the treadmill in a situation similar to what they had experienced during the familiarization trials. To facilitate participant's visualization of slipping while walking, the videos included the posterior (back) view of a person walking, or slipping while walking on the treadmill. All the imagined and observed tasks were performed before and after perturbation training. During the MRI session, subjects laid still in the MR scanner and were instructed to perform two different mental imagery tasks. While in the MR scanner, participants alternated between four tasks—(1) mental imagery of themselves slipping while walking on the treadmill (imagined slipping), (2) mental imagery of themselves walking on the treadmill (imagined walking), (3) observation of another person slipping while walking on the treadmill, and (4) observation of another person walking on the treadmill. These experimental tasks were interspersed with periods of rest. The duration of mental imagery, observation, and rest was 30 s. All tasks were presented in a randomized order. Each participant performed two blocks of two trials for each condition. We presented auditory cues to indicate the start and end of metal imagery tasks. At the beginning of mental imagery tasks, participants received the auditory cue “close your eyes and imagine yourself walking or slipping while walking on the treadmill.” At the end of mental imagery tasks, participants received the auditory cue “open your eyes” to proceed to the next fMRI task. In the rest condition, participants were instructed to focus on “X” sign presented on the screen, trying not to think about anything else. After each MRI session, we surveyed the participants for the number of slips imagined in the imagined slipping condition.

### Behavioral Data Acquisition and Analysis

Full body kinematics were recorded using an eight camera 3D motion capture system (Motion Analysis, Corp, Santa Rosa, California). The Helen Hayes marker set comprising of 29 passive markers placed on the bony landmarks of bilateral upper extremities and lower extremities, trunk and head was used to compute the joint centers and center of mass (COM) (34). The motion capture data was collected at a sampling rate of 120 Hz. The raw marker data were low-pass filtered through fourth-order Butterworth filter with a cut-off frequency of 6 Hz. A load cell connected in series with the harness measured the amount of body weight supported by the harness during each trial. The kinematic variables were computed using a custom-written algorithm in MATLAB (version 2014b, The Mathworks Inc, Natick, MA, USA).

### Behavioral Outcomes

To examine adaptation to slip perturbation training we measured the following outcomes measures: (i) perturbation outcome, (ii) number of compensatory steps, and (iii) COM stability.

Perturbation outcome: For each perturbation trial, we determined whether the participant experienced a fall or showed a recovery response. The trial was identified as a fall if it was apparent that the participant was supported by the harness and the force exerted on the load cell exceeded 30% of the body weight for >1 s after perturbation onset (35), or when the subjects failed to initiate a stepping response resulting in a catch by the harness. The remaining trials were identified as recoveries.

#### Compensatory Step

The number of steps taken between perturbation onset (phase C–E in Figure 2A) to perturbation termination were counted. The first compensatory step was described as a step taken with the trailing limb and landing behind the slipping limb. The subsequent steps could be taken with the slipping or the trailing limb; however, always landing behind the contralateral limb. The compensatory stepping phase was also termed the loss of balance phase. If subjects were able to successfully take a forward step in presence of the perturbation it was considered a regular step with no balance loss. The Z-coordinate of the stepping limb heel marker was used to identify the unloading of the foot.

#### Stability

The center of mass (COM) position was calculated relative to the rear of the base of support (BOS) i.e., heel of the most posterior limb (slipping limb during single leg stance at liftoff of the trailing limb and trailing limb at step touchdown). The COM position was then normalized by the participant's foot length. The COM velocity was obtained by the first order differentiation of the COM position. The COM velocity was calculated relative to the heel velocity of the most posterior limb (similar to COM position) and normalized by a dimensionless fraction of √g^*^h, where g denotes gravity and h denotes the individual's body height. The COM stability was computed as the shortest distance of the instantaneous COM state (position and velocity) with respect to the theoretical boundary for backward loss of balance (36, 37). A negative stability value indicated by a COM state below the threshold boundary signifies a greater predisposition for backward balance loss. If the stability value is 0 it indicates that the COM state lies on the computational boundary. While positive stability values signify recovery from loss of balance indicating the COM state lies within the boundary (Figure 2C). Stability values were obtained at touchdown of the first compensatory step.

### fMRI Data Acquisition

Whole brain imaging was performed with a 3.0 T GE Discovery scanner (Milwaukee, WI) using a standard radio frequency coil and T2^*^-weighted pulse sequence. BOLD functional images were collected using a gradient-echo axial echo planar imaging sequence (38) at University of Illinois at Chicago. The following parameters were used: repetition time = 2,000 ms, echo time = 22.2 ms, flip angle = 90 degree, 64 by 64 parcellated matrix of 220 mm by 220 mm field of view, slice thickness = 3 mm, 44 slices, and voxel size of 3.4 mm by 3.4 mm. The repetition time is defined as the time between the beginning of two consecutive pulse sequences, the echo time is the time between the center of exciting *RF* pulse and the center of spin echo, and the field of view (FOV) is defined as the rectangular region over which the MRI image is acquired. The matrix size refers to the parcellations of the field of view. The matrix size determined the size of the voxels contained in the FOV. An axial T1 spoiled gradient structural image was obtained for each using 182 axial images and 1 mm in thickness for spatial normalization [minimum TR/TE (9.292 ms/3.77 ms) TI = 450 ms]. During scanning, participants completed the observed and imagined tasks. Prior to scanning, the importance of remaining motionless was conveyed to each participant. There were two runs of the experimental tasks, each lasting 5 min and 20 s, and acquiring 120 volumes. Overall, across the two runs, each of the mental imagery, observation and rest tasks were performed four times. Each condition was averaged across both runs, prior to the creation of subtraction contrast analyses.

### fMRI Preprocessing

Images underwent slice-timing corrections with SPM8 (http://www.fil.ion.ucl.ac.uk/spm/doc/) and motion detection algorithms with FSL (http://fsl.fmrib.ox.ac.uk/fsl/fslwiki/). During pre-processing, images were visually inspected for motion >1.5 mm across more than three TRs. This motion check did not result in the exclusion of any participants from analyses. Structural and functional images were co-registered and then the co-registered T1-SPGR underwent spatial normalization (DARTEL to MNI template). The resulting normalization matrix was then applied to the slice-time-corrected, movement- adjusted time series data and smoothed with a 5 mm Gaussian kernel. Resulting T2^*^ images were 2 mm on a side with isotropic voxels.

### Statistical Analysis

The effect of perturbation training was analyzed by comparing the number of compensatory steps and COM state stability at touchdown between the first trial of first training session (day 1) and the last trial of last training session (day 3) for low, mid and highest perturbation levels (I, III, and VI). The change perturbation outcome and in number of compensatory steps between day 1 and day 3 was compared using the Wilcoxon Signed Rank test. The effect of perturbation intensity on perturbation outcome and number of steps was examined by Kruskal–Wallis test. A significant main effect was then followed up by Mann-Whitney U test for comparison between the perturbation intensities. The changes in COM state stability at touchdown was examined by 2 × 3 two-way analysis of variance (ANOVA) with stability at touchdown as dependent variable and perturbation intensity (I, III, and VI) and training sessions (day 1 and day 3) as independent variables. *Post hoc* paired and independent *t-*tests were performed to resolve any significant main effects and interactions. All 10 participants completed the three consecutive treadmill-slip perturbation training sessions. Therefore, all analyses were performed on 10 participants. The significance level was set at *p* < 0.05.

With regards to fMRI data analysis, we compared changes in neural activation before and after perturbation training only in the imagined conditions. Our recent study demonstrated that mental imagery of slipping induced greater engagement (corresponding with more areas of activation) of neural structures than the observation of slipping. There was no difference in neural activation between mental imagery and observation of regular walking (39). Based on these recent findings, in current study, we considered only the imagined conditions for comparing pre- to post-perturbation training fMRI changes. First, activation during rest in the pre-training scan was subtracted from activation during the experimental conditions in the pre-training scan to create two baseline contrasts of interest: baseline IS minus rest and baseline IW minus rest. Second, activation during rest in the post-training scan was subtracted from activation during the experimental conditions in the post-training scan to create two post-training contrasts of interest: post IS minus rest and post IW minus rest. Then, using a within-group ANOVA in SPM8, activation during the post-training scan was compared to pre-training activation by subtracting the respective contrasts of interest from each scan, e.g., baseline IS compared to post-training IS. We also examined the difference between post-training activations in the IS and IW conditions. A gray matter mask was applied and whole brain alpha of .01 was achieved for each contrast. The default familywise error option in SPM was not used. Instead, cluster extent was determined based upon 1000 Monte Carlo simulations in the bug-fixed 3dClustSim tool (40), resulting in a joint threshold of height and extent (*p* < 0.01, extent of 640 mm^3^ or k = 80 voxels). The Monte Carlo approach was intended to balance Type I and Type II error.

## Results

### Adaptation to Treadmill-Slip Perturbations

All participants upon perturbation onset (Figure 2C, star) experienced loss of balance observed by initiation of a backward compensatory step, with the COM state traveling below the computational backward balance loss threshold (Figure 2C, square) in response to treadmill-slip perturbation at all perturbation intensities. Participants demonstrated a compensatory step for recovery from balance loss resulting in a positive stability with the COM state lying above the threshold for backward balance loss (Figure 2C, circle). With regards to perturbation outcome, there was no difference in falls incidence on level I perturbations between day 1 (0%) and day 3 (0%) (*p* > 0.05). Similarly, there was no change in falls incidence on level III perturbations between day 1 (10%) and day 3 (0%) (*p* > 0.05). For level VI perturbation intensity, the incidence of falls significantly reduced from day 1 (60%, 6/10) to day 3 (0%) (*Z* = −2.44*, p* < 0.05).

**Figure 3:**
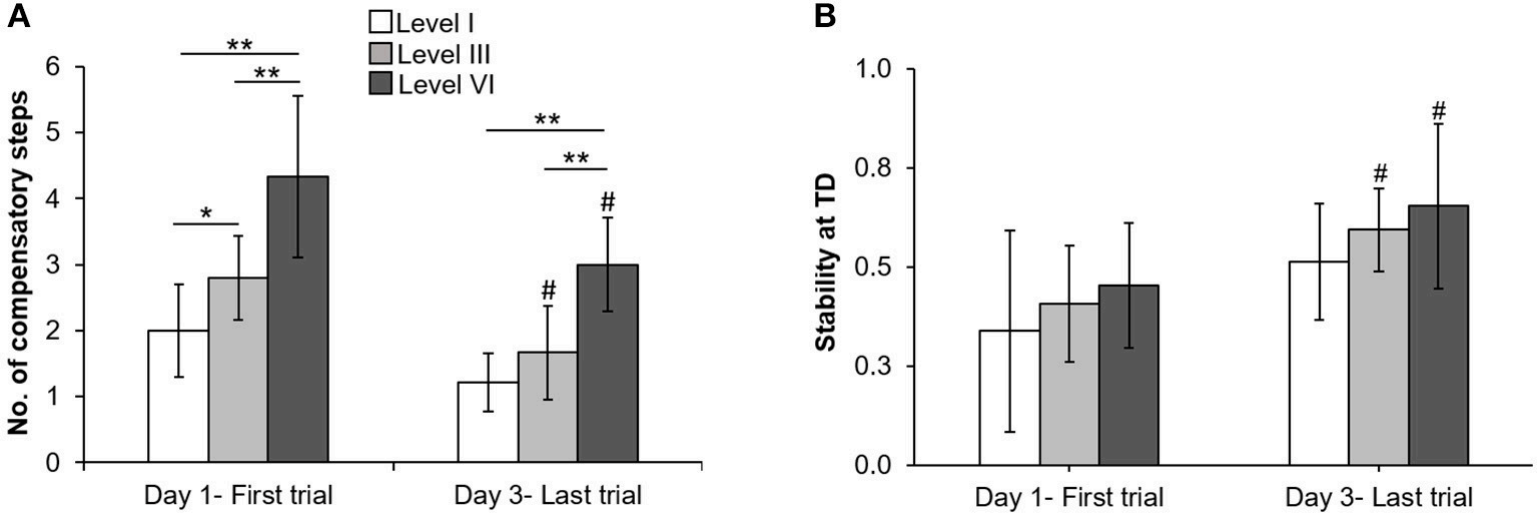
Changes in mean (±SD) **(A)** number of compensatory steps after perturbation onset and **(B)** COM state stability at compensatory step touchdown (TD) at lowest (level I), mid (level III), and highest (VI) perturbation intensities between first training trial on day 1 and last training trial on day 3. ^*^p < 0.05 and ^**^p < 0.01 indicates significant differences between different perturbation intensities on the same day and ^#^*p* < 0.05 indicates significant differences between same intensities on first (day 1) and last day (day 3).

On the lowest perturbation intensity (level I), there was no change in number of compensatory steps from day 1 to day 3 (*Z* = −1.89, *p* > 0.05, Figure 3A). The number of compensatory steps significantly reduced on the level III (*Z* = −2.33, *p* < 0.05) and level VI (*Z* = −2.15, *p* < 0.05) perturbations on day 3 compared with day 1. There was a significant main effect of perturbation level on number of steps. More number of steps were observed at higher perturbation intensities for day 1(χ^2^ = 14.32, *p* < 0.01) and day 3 (χ^2^ = 15.91, *p* < 0.01). For COM state stability at compensatory step touchdown, comparison of the training session (day 1 and day 3) across the three perturbation intensities (levels I, III, and VI) showed no significant training session × level interaction *F*_(2, 24)_ = 0.06, *p* > 0.05, Figure 3B. There was a significant main effect of training session *F*_(1, 24)_ = 10.67, *p* < 0.01, partial η^2^ = 0.30 and a significant main effect of perturbation intensity *F*_(2, 24)_ = 3.60, *p* < 0.05, partial η^2^ = 0.23. On lowest perturbation intensity (level I), there was no significant difference in stability at touchdown from day 1 and day 3 (*p* > 0.05). On level III & VI perturbation intensity, the stability at touchdown significantly increased from day 1 to day 3 (*t* = −2.80, *p* = 0.01 for level III, *t* = −2.07, *p* = 0.03 for level VI).

### Changes in Neural Activation With Behavioral Adaptation

All participants reported the ability to form a mental image of a motor task prior to performing the fMRI tasks, with a median score of 4.5/5 on vividness of visual imagery of questionnaire. During both MRI sessions, the participants imagined 3–5 slips in the imagined slipping condition. At baseline, prior to treadmill-slip perturbation training imagined slipping and imagined walking demonstrated increased activation in several cortical and subcortical areas (Table 1). As compared with the rest condition, imagined slipping yielded activation in the frontal, parietal, and limbic regions including superior frontal gyrus (SMA, BA6), inferior frontal gyrus, inferior parietal lobule, parahippocampal gyrus, cingulate gyrus, and posterior cerebellum (*p* < 0.01, Table 1). Imagined walking demonstrated an increased activation in the left medial frontal gyrus (BA 32), left precentral gyrus, and right inferior frontal gyrus (*p* < 0.01, Table 1).

**Table 1:** Differences in brain activation between imagined slipping, imagined walking, and rest conditions in the pre-training fMRI session.

**Contrast**	**Anatomical regions**				**Voxels**	**MNI coordinates**			
	**Lobe**	**Gyrus**	**BA**	**Side**		**x**	**y**	**z**	***Z*-value**
IS vs. Rest	Frontal	Superior frontal gyrus	6	L	130	−32	12	54	3.80
		Inferior frontal gyrus	45	R	475	56	10	22	3.92
	Parietal	Inferior parietal lobule	40	L	115	−46	−46	46	3.48
	Limbic	Parahippocampal	–	R	123	14	−10	−18	3.34
		Cingulate gyrus	24	L	3716	−4	−8	50	4.07
	Cerebellum	Posterior lobe-declive	–	R	210	28	−74	−28	3.77
				L	184	−38	−60	−30	3.54
IW vs. Rest	Frontal	Medial frontal gyrus	32	L	162	−6	6	50	3.49
		Precentral	–	L	100	−48	6	14	3.53
		Inferior frontal gyrus	44	R	95	54	10	20	3.11

*IS, Imagined slipping; IW, Imagined walking; MNI, Montreal Neurological Institute; BA, Broadmann area; SMA, Supplementary motor area. All activations were observed at p < 0.01 and k = 80*.

**Figure 4:**
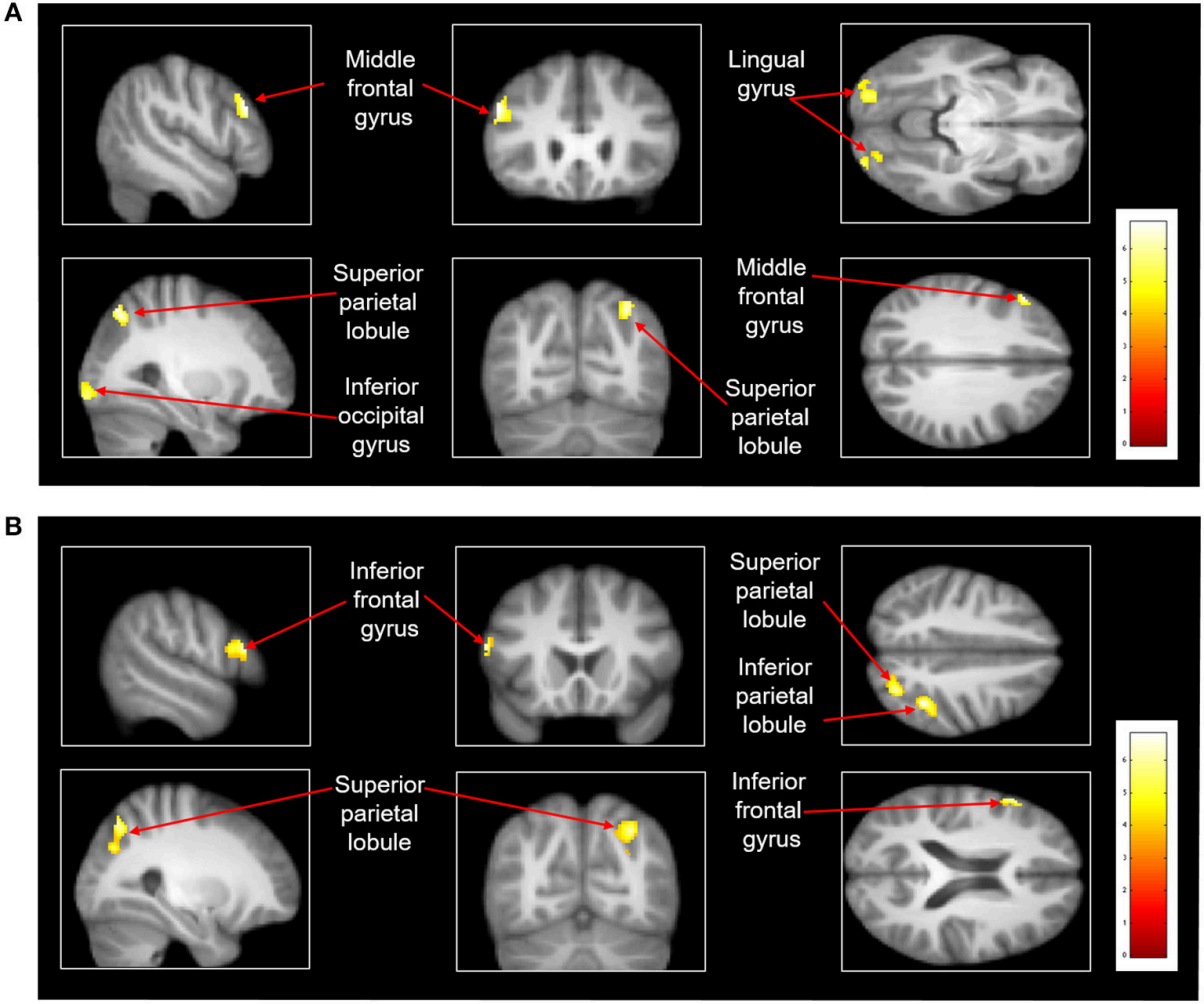
Heat map demonstrating areas with greater activation in the **(A)** imagined slipping condition and **(B)** imagined walking condition after 3 consecutive days of treadmill-slip perturbation training. Increased activation was noted in different regions in the frontal, parietal, and occipital lobes (*p* < 0.01, k = 80).

After treadmill-slip perturbation training, there was an increased activation in several cortical regions in the imagined slipping condition (Table 2). These include left middle frontal gyrus (dorsolateral prefrontal cortex, BA 9), right superior parietal lobule (BA 39), right inferior occipital gyrus (BA 18), and left lingual gyrus (BA 18) (*p* < 0.01). The heat map showing greater activation in cortical areas post-training compared with pre-training is shown in Figure 4A. None of the brain areas showed a decrease in activation post-training in the imagined slipping condition. Treadmill-slip perturbation training also influenced the brain activation in the imagined walking condition. There was an increased activation in the frontal, parietal, and occipital regions (*p* < 0.01), including left inferior frontal gyrus (BA 44), right inferior parietal lobule (BA 40), and right superior parietal lobule (BA 39) in the imagined walking condition (Figure 4B, Table 2). There was no significant decrease in activation in any of the brain regions post-training (*p* > 0.01). Further, a comparison of post-training imagined slipping and post-training imagined walking conditions revealed significant differences in cortical and subcortical activations between the two conditions (*p* < 0.01). Specifically, the bilateral anterior cerebellum, bilateral posterior cerebellum, superior and middle temporal gyrus, right middle frontal gyrus (BA 10), left supplementary motor area (BA 6), left precuneus (BA 31), anterior cingulate (BA 25), and posterior cingulate (BA 23), and left parahippocampal gyrus showed increased activation in post-training imagined slipping compared with post-training imagined walking condition (Table 3).

**Table 2:** Differences in pre and post treadmill-slip perturbation training brain activation in imagined slipping and imagined walking conditions.

**Contrast**	**Anatomical region**				**Voxels**	**MNI coordinates**			
	**Lobe**	**Gyrus**	**BA**	**Side**		**x**	**y**	**z**	***Z*-value**
IS	Frontal	Middle frontal gyrus	9	L	190	−48	26	30	3.44
	Parietal	Superior parietal lobule	39	R	87	30	−68	46	3.30
	Occipital	Inferior occipital gyrus	18	R	174	32	−92	−6	3.12
		Lingual gyrus	18	L	226	−22	−90	−10	3
IW	Frontal	Inferior frontal gyrus	44	L	140	−56	18	18	3.52
	Parietal	Inferior parietal lobule	40	R	201	38	−46	44	3.75
		Superior parietal lobule	39	R	268	28	−66	44	3.42

*IS, Imagined slipping; IW, Imagined walking; MNI, Montreal Neurological Institute; BA, Broadmann area. All activations were observed at p < 0.01 and k = 80*.

**Table 3:** Differences in brain activation between post perturbation training imagined slipping and post perturbation training imagined walking conditions.

**Contrast**	**Anatomical region**				**Voxels**	**MNI coordinates**			
	**Lobe**	**Gyrus**	**BA**	**Side**		**x**	**y**	**z**	***Z*-value**
IS vs. IW	Frontal	Medial frontal gyrus	10	R	1182	8	56	−4	4.56
		Superior frontal gyrus	6	L	125	−6	8	64	3.16
	Parietal	Precuneus	39	L	203	−40	−74	36	3.21
	Temporal	Superior temporal gyrus	38	L	225	−50	8	−12	3.16
		Middle temporal gyrus	39	R	107	54	−66	26	3.14
	Limbic	Posterior cingulate	31	L	1871	−8	−58	24	3.73
		Anterior cingulate	25	L	83	−6	20	−2	3.35
		Parahippocampal	–	R	105	24	−30	−20	3.30
	Cerebellum	Anterior lobe	–	R	182	14	−46	−30	3.62
			–	L	182	−8	−46	−28	3.21
		Posterior lobe	–	L	372	−8	−64	−24	3.33
			–	R	107	26	−76	−22	3.07

*IS, Imagined slipping; IW, Imagined walking; MNI, Montreal Neurological Institute; BA, Broadmann area. All activations were observed at p < 0.01 and k = 80*.

## Discussion

The current study provides evidence regarding the specific neural structures associated with mental imagery of slipping after adaptation to sudden slip-like perturbations while walking. Over a period of 3 consecutive days, the participants adapted to small and large magnitude treadmill-slip perturbations showing reduced incidence of falls and increased COM state stability at compensatory step touchdown on day 3 compared with day 1. Behavioral adaptation was accompanied by increased activation in several cortical and subcortical areas in the imagined slipping and imagined walking conditions. Finally, post-training imagined slipping showed greater engagement of motor, sensory, limbic, and cerebellar areas compared with post-training mental imagery of regular walking.

A general baseline activation was observed in SMA (superior frontal gyrus), inferior frontal gyrus, inferior parietal lobule, parahippocampal gyrus, and cingulate gyrus during imagined slipping (Table 1). But following 3 consecutive days of treadmill-slip perturbation training, we identified increased activity predominantly in the frontal and parietal cortical areas in the imagined slipping condition. These findings suggest a significant role of frontal and parietal cortical structures in learning a relatively novel, less practiced postural task related to perturbed locomotion. Behavioral adaptation to perturbations was evident on mid (level III) and highest (VI) perturbation intensities as the stability at compensatory step TD increased from day 1 to 3 reflecting that the participants acquired the ability to maintain a more anterior i.e., more stable COM state following perturbation. Thus, with repeated exposure to such perturbations, the reactive response was refined to achieve an improved dynamic stability control against slip-like perturbations.

We also found significant changes in neural activation evidenced by increased recruitment of dorsolateral prefrontal cortex (DLPFC, middle frontal gyrus, BA 9) after 3 consecutive days of perturbation training. Learning a new task requires attention which would be particularly higher in the early phase when the CNS is forming the association between the tasks details and the necessary responses (41). Thus, DLPFC which plays a key role in decision making and working memory (42), is likely recruited to temporarily store the sensorimotor and joint position information following each perturbation, to inhibit the unwanted response and carry out the perturbation specific response, as the CNS becomes progressively more aware of perturbation characteristics. Prefrontal cortex activation has been observed during tasks requiring greater balance demands rather than during static standing or regular walking e.g., walking on a narrow pathway (40) and heel-to-toe walking (14). Thus, increased activation in DLPFC during imagined slipping after training could suggest that significant attention is required to recover balance from sudden slip-like perturbations.

DLPFC is also implicated to play a role in learning novel tasks. It is particularly activated when performing tasks that involve learning new motor sequences (41, 43). It is postulated that with repeated perturbation exposure the CNS updates its internal representation of stability limits with the sensory feedback of previous trials and subsequently builds a motor repertoire. On subsequent exposure to a similar perturbation the CNS can trigger the motor repertoire with greater stability and resulting recovery strategies (36, 44, 45). Few studies have shown cortical modulation during reactive balance response preparation and execution even for the very first novel recovery response, primarily through involvement of prefrontal cortex, premotor and parietal areas (26, 27, 46). In our study, greater activation in DLPFC during imagined slipping after training indicates a potential role of the prefrontal lobe in developing the internal representation of the motor response for a relatively novel balance task. Although there is evidence that DLPFC activation reduces after the initial motor sequence learning phase (43), we found increased activation in DLPFC in the post-training compared with pre-training imagined slipping. It is likely that such increase in prefrontal activation with a short training period may decline with further training or time lapse due to the process of consolidation as the behavioral learning response stabilizes.

Adaptation to treadmill-slip perturbations also showed increased activation in right superior parietal lobe (BA39) and BA 18 (right inferior occipital gyrus & left lingual gyrus) during imagined slipping. Activation in these areas suggests greater engagement of the association areas in the parietal and visual regions while learning complex postural tasks. Association areas seem to be involved in higher order processing of information rather than only identifying simple characteristics of the sensory input (47). With repeated exposure to varied intensities of slip-like perturbations, association areas perhaps combine the visual and kinesthetic feedback for faster processing of visuo-spatial information to aid decision making. Such involvement of visual and parietal association areas is consistent with adaptation to visuomotor tasks. Studies have shown activation of parieto-occipital sulcus while learning visuomotor tasks (48–50). Further, there is contribution of inferior occipital and lingual gyrus (BA 18) during adaptation and transfer of learned visuomotor tasks to the untrained hand (51). Considering that these areas did not show activation at baseline (before training) but showed increased activation post-training, suggests that the association areas could play a significant role in acquisition of movements requiring visuomotor coordination.

Limited studies have examined changes in brain activation pattern with adaptation of locomotor balance tasks. Ionta et al. examined cortical plasticity after a single session of treadmill-delivered locomotor-balance training via a mental imagery paradigm. They observed increased activation in SMA, thalamus, right basal ganglia, and right cerebellum during the imagined walking condition, after 20 min of walking practice (52). Our study extends findings to balancing during a locomotor task showing recruitment of specific brain regions after locomotor-balance training sessions. However, unlike Ionta et al. (52) our study showed increased activation post-training predominantly in the cortical regions. Such differences in current and previous findings could be related to the walking task. Ionta et al. (52) examined pre to post neural changes after regular walking, a task relatively more practiced task than perturbed walking. The authors suggest that increased recruitment of lower brain centers (subcortical regions) could be related to maintaining sequence, timing, and coordination of limb movements rather than recruiting higher centers to establish a new internal presentation of body schema. On the other hand, increased recruitment of higher brain centers (cortical areas) in our study could be related to processing of novel somatosensory stimuli to learn and execute a different motor response than that involved in regular walking.

Before training, in the imagined slipping condition, activations were observed in SMA, parahippocampal gyrus, cingulate gyrus, and posterior cerebellum (see Table 1) which was not modulated (neither increased nor decreased) through adaptive perturbation training (see Table 2). These findings suggest that the above areas could be consistently activated pre- and post-training in balance recovery from treadmill-slip perturbations. Activation in these brain centers also represents recruitment of indirect neural pathways, predominantly responsible for planning and modulation of movements during locomotor tasks (11). This idea is also supported by another study showing increased activation of SMA and parahippocampal gyrus during imagined locomotor task which involved navigating around a series of obstacles (53). Similarly, in our study, repeated exposure to external perturbations would require extensive planning based on prior exposures, monitoring of ongoing movements at the time of perturbation, and executing a planned response.

Locomotor treadmill-slip perturbation training also resulted in an increased activation in the parietal regions during imagined walking. The 3-day training task although was targeted to improve reactive balance, it also provided substantial walking exercise and hence could have provided some locomotor training itself. Further, the subjects who participated in training were asked not to perform any form of locomotor exercise training (treadmill walking, running, bicycling etc.) during the perturbation training period. Secondly, considering that both imagined conditions involved walking, it is likely that both conditions could share similar neural pathways and therefore resulted in increased activation in some common cortical areas after perturbation training. Nevertheless, we found that after perturbation training, the brain activity differed between imagined slipping and walking conditions.

Post-training, imagined slipping compared with imagined walking yielded greater activity in medial and superior frontal, parietal, cingulate, parahippocampal, and cerebellar regions. Some of these regions also demonstrate increased activation in imagined-slipping post-perturbation training, suggesting that changes in neural activation pre- and post- imagined slipping could be related to slip-perturbation training. In contrast to regular walking, slipping introduces movement errors in walking that need corrective responses and therefore, possibly exerts higher cognitive load for balance control. Accordingly, we found stronger brain activity in post-training imagined slipping vs. post-training imagined walking. The slip-perturbation training resulted in improved reactive postural stability through feedback learning which involved mapping of movement errors and updating the resultant motor responses. Regions such as medial frontal region, anterior cingulate, and cerebellum are associated with motor error detection and error-processing within the feedback control (54–56). These regions were particularly more active in post-training imagined slipping than post-training imagined walking, explaining that potentially different brain regions are engaged in learning reactive balance strategies in walking than regular walking.

Previous studies using functional imaging through mental imagery, electroencephalography, and functional near-infrared spectroscopy report a predominance of activity in left pre-frontal, frontal, and parietal regions during a relatively complex locomotor tasks (e.g., increasing walking speed, walking while talking, walking on a balance beam, and external perturbations in standing), suggesting that the left sensorimotor areas play a role in executing complex and skilled movements in locomotion (13, 14, 57, 58). Our findings concur with previous studies to some extent such that post-training, we found increased activation in left frontal areas in both the metal imagery conditions and increased activation in left fronto-parietal regions in post-training imagined slipping verses imagined walking.

Given that the evidence regarding neural pathways related to reactive balance is still emerging, this study provides novel insights into the specific brain areas involved in learning a locomotor reactive balance task in healthy young adults. Our findings should however be interpreted in light of some limitations. An important limitation of the study includes use of mental imagery instead of real locomotion. While there is some evidence regarding overlapping brain activity between mental imagery and overt movements (11, 59) the mental imagery of slipping while walking may not emulate the sensorimotor experience of actual movements. Further, it is difficult to ensure consistency in mental imagery across participants. Although we screened for the ability to perform mental imagery prior to MR scans, adherence to mental imagery during the scan is difficult to assess. We however decided to incorporate the fMRI approach based on the extensive evidence supporting use of mental imagery for examining neural activity of locomotor tasks (13, 15, 60) and to achieve better spatial resolution compared with other more portable imaging techniques like fNIRS or EEG (61). Additionally, the study design did not allow for identification of changes in neural activity during the exact instant when the participants imagined slipping. Therefore, we included the imagined walking as a control condition to be able to compare the differences, if any, in neural activity related to perturbed walking practice vs. regular walking practice. Due to lack of research on neural changes involved in adaptation to locomotor balance tasks, this study was designed as a proof of concept study and is therefore limited by a small sample size. While our study provides proof of feasibility, future studies with a larger sample size are recommended. Future investigations should also compare neural activation between perturbation-training and regular walking training for further insight into neural mechanisms specific to reactive balance adaptation.

This fMRI study identified a specific brain areas involved in learning of a locomotor reactive balance task involving balance recovery during slip-like external perturbations. The findings support that learning challenging balance tasks requiring precise and coordinated movements is associated increased activity in DLPFC, parietal, and visual association areas. In addition, there is continued activation of the parahippocampal and cingulate regions which assists in the learning process.

## Ethics Statement

The study procedures were carried out in accordance with the recommended guidelines of the University of Illinois at Chicago Institutional Review Board. All participants were enrolled into the study after obtaining an informed written consent. The protocol was approved by University of Illinois, Biomedical and Biological Sciences Board.

## Author Contributions

All authors contributed significantly toward study execution and manuscript preparation. TB contributed toward conceptual idea/research design. TB and PP contributed toward project management, data collection, data analysis, and manuscript preparation. SL and SRD provided consultation for research design and data analysis. SD assisted with data analysis, data processing, and reviewing manuscript. TB provided research funding, equipment, and facilities for perturbation training.

### Conflict of Interest Statement

The authors declare that the research was conducted in the absence of any commercial or financial relationships that could be construed as a potential conflict of interest. The reviewer CP and handling Editor declared their shared affiliation at the time of the review.
